# Mapping hospital data to characterize residents’ educational experiences

**DOI:** 10.1186/s12909-022-03561-x

**Published:** 2022-06-25

**Authors:** David W. Rhee, Ilan Reinstein, Morris Jrada, Jay Pendse, Patrick Cocks, David T. Stern, Daniel J. Sartori

**Affiliations:** 1 Leon H. Charney Division of Cardiology, Department of Medicine, NYU Grossman School of Medicine, New York, NY USA; 2 Institute for Innovations in Medical Education, NYU Langone Health, New York, NY USA; 3 Department of Medicine, NYU Grossman School of Medicine, New York, NY USA; 4 Division of Endocrinology, Department of Medicine, NYU Grossman School of Medicine, New York, NY USA; 5 Internal Medicine Residency Program, Department of Medicine, NYU Grossman School of Medicine, NYU Langone Hospital – Brooklyn, New York, USA; 6 VA NY Harbor Health, New York, NY USA

**Keywords:** Content mapping, Precision education, Rational curriculum design

## Abstract

**Background:**

Experiential learning through patient care is fundamental to graduate medical education. Despite this, the actual content to which trainees are exposed in clinical practice is difficult to quantify and is poorly characterized. There remains an unmet need to define precisely how residents’ patient care activities inform their educational experience.

**Methods:**

Using a recently-described crosswalk tool, we mapped principal ICD-10 discharge diagnosis codes to American Board of Internal Medicine (ABIM) content at four training hospitals of a single Internal Medicine (IM) Residency Program over one academic year to characterize and compare residents’ clinical educational experiences. Frequencies of broad content categories and more specific condition categories were compared across sites to profile residents’ aggregate inpatient clinical experiences and drive curricular change.

**Results:**

There were 18,604 discharges from inpatient resident teams during the study period. The crosswalk captured > 95% of discharges at each site. Infectious Disease (ranging 17.4 to 39.5% of total discharges) and Cardiovascular Disease (15.8 to 38.2%) represented the most common content categories at each site. Several content areas (Allergy/Immunology, Dermatology, Obstetrics/Gynecology, Ophthalmology, Otolaryngology/Dental Medicine) were notably underrepresented (≤ 1% at each site). There were significant differences in the frequencies of conditions within most content categories, suggesting that residents experience distinct site-specific clinical content during their inpatient training.

**Conclusions:**

There were substantial differences in the clinical content experienced by our residents across hospital sites, prompting several important programmatic and curricular changes to enrich our residents’ hospital-based educational experiences.

**Supplementary Information:**

The online version contains supplementary material available at 10.1186/s12909-022-03561-x.

## Background

Experiential learning through patient care is a cornerstone of graduate medical education (GME). While GME curricula comprise other important modes of learning – for example structured didactic programs and guided self-study, which enrich specific content and supplement trainee’s clinical exposure – it is active participation in real-life care of patients that forms the bulk of trainees’ education and drives their development as physicians [1].

Despite this, the medical content to which trainees are exposed though their clinical experiences is difficult to quantify and remains poorly characterized. While training programs seek to expose their trainees to both an appropriate volume as well as diversity of clinical conditions, little is known about whether these are achieved, relative to peer programs or governing bodies’ expectations. The distribution of medical content reflected by trainee’s clinical practice likely varies widely across training programs, owing to individual programs’ distinct clinical services, patient populations, and educational focus. Even within a single program, the experiences of trainees are subject to significant variability [2], and this is amplified for larger programs whose trainees rotate across diverse training sites. Importantly, variability in clinical exposure may underlie differences in trainees’ clinical strengths or weaknesses, and may predict competency or even future career choice among trainees [3–6].

Thus, to fully understand and help shape the education and trajectory of their trainees, graduate medical educators need to have a comprehensive understanding of their clinical experiences. We have recently developed a crosswalk tool to augment our ability to interpret Internal Medicine (IM) residents’ clinical experiences [7, 8]. This strategy, modeled after that introduced by Gray and colleagues [9] maps resident-attributed ICD-10 diagnosis codes to American Board of Internal Medicine (ABIM) content categories and effectively translates residents’ clinical experiences into a common educational language. We have piloted this strategy at a single training site within the NYU Internal Medicine Residency Program and mapped a sample of residents’ inpatient experiences to ABIM content areas, yielding a useful clinical content profile.

However, our IM Residency Program, one of the largest in the country, comprises well over 200 residents who rotate across four distinct hospital systems, exposing residents to tremendously diverse, and presumably variable clinical content. A detailed characterization of the clinical content seen at each training site could uncover important differences in the clinical experiences of our trainees, and could drive evidence-based curricular changes aimed at bolstering exposure to certain content areas and perhaps reducing exposure to others to meet residents’ needs. Importantly, employing this strategy across an especially broad scope of practice sites – namely a community-based university hospital, a large quaternary care hospital center, a public city hospital, and a federal Veterans Affairs hospital – could serve as a generalizable model for other training programs to pursue similar analyses.

In this study, we translate resident-attributed principal ICD-10 discharge diagnosis codes from each of our program’s four training hospitals throughout academic year 2020–2021 to quantify and compare the clinical-educational experiences of our residents, test for differences in exposure among the hospitals, and drive evidence-based curricular change.

## Methods

### Setting and participants

At the time of this study, the NYU IM Residency Program comprised 221 residents (64 PGY1, 29 Preliminary PGY1, 65 PGY2, 63 PGY3) whose inpatient medicine experience took place across four hospitals: NYU Langone Hospital – Brooklyn (NYU-BK; an academic community hospital), NYU Langone Hospitals – Manhattan (NYU-MN; a large university-based quaternary care hospital), Bellevue Hospital (BH; a large public hospital), and VA NY Harbor Healthcare – Manhattan (VA; a federal Veteran’s Affairs Hospital). Inpatient rotations constitute roughly two-thirds of total training time and site assignments are weighted by training track, such that cohorts of residents complete the majority of inpatient training at a single site. During the study period, there were 7 total inpatient (acute care and intensive care) teams at NYU-BK, 7 at NYU-MN, 12 at BH, and 4 at VA.

### Measures

We mined principal ICD-10 discharge diagnosis codes from all resident-staffed acute care and intensive care inpatient teams at each hospital over one academic year (July 1, 2020 to June 30, 2021), which were made available through each site’s data analytics team. Of note, principal ICD-10 diagnosis codes, which describe the condition that occasioned hospital admission, are routinely assigned in standardized fashion by hospital coders after patient discharge and do not reflect individual coding decisions by residents or attending physicians.

We have previously described the development of a crosswalk tool, a repository of ~ 5000 ICD-10 codes anchored to 16 broad medical content categories and 177 more specific condition categories to characterize the inpatient teaching service at NYU-BK [7]. One-hundred-and-four additional ICD-10 codes from BH, NYU-MN, and VA were categorized by three blinded adjudicators and added to the table to ensure that it reliably captured > 95% of ICD-10 codes from each of the four sites (Supplemental Table 1). Diagnosis codes were deemed “captured” by the crosswalk if the syntax of an ICD-10 code in the crosswalk table exactly matched or was nested within the ICD-10 code in question. For example, the hypothetical diagnosis code “X50.2” would capture “X50.2”, “X50.20”, “X50.21.” Principal, and not secondary, ICD-10 codes were used given that they define the singular compelling reason for hospitalization and thus were deemed to carry the educational weight of an admission. This approach excludes extraneous diagnoses often contained in lists of secondary ICD-10 codes assigned to patients, and is consistent with prior attempts to map inpatient diagnoses [2 , 8, 10].

**Table 1:** Frequencies of patient discharges mapping to sixteen content categories across the four training sites of the NYU Internal Medicine Residency Program

Medical Content Category	NYU-BK	NYU-MN	BH	VA	*p*-value
Infectious Diseases	2485 (39.5)	1501 (30.8)	1088 (20.6)	256 (17.4)	< 0.001
Cardiovascular Disease	995 (15.8)	1243 (25.5)	1631 (31.0)	562 (38.2)	< 0.001
Gastroenterology	593 (9.4)	511 (10.5)	425 (8.1)	124 (8.4)	< 0.001
Pulmonary Disease	364 (5.8)	300 (6.2)	216 (4.1)	121 (8.2)	< 0.001
Neurology	301 (4.8)	178 (3.7)	402 (7.6)	126 (8.6)	< 0.001
Hematology	186 (3.0)	255 (5.2)	286 (5.4)	43 (2.9)	< 0.001
Psychiatry	336 (5.3)	104 (2.1)	355 (6.7)	48 (3.3)	< 0.001
Endocrinology Diabetes and Metabolism	353 (5.6)	181 (3.7)	201 (3.8)	48 (3.3)	0.0024
Nephrology and Urology	331 (5.3)	197 (4.0)	172 (3.3)	43 (2.9)	< 0.001
Medical Oncology	118 (1.9)	174 (3.6)	242 (4.6)	30 (2.0)	< 0.001
Rheumatology and Orthopedics	129 (2.1)	126 (2.6)	129 (2.4)	44 (3.0)	0.0207
Obstetrics and Gynecology	38 (0.6)	48 (1.0)	36 (0.7)	3 (0.2)	0.6067
Otolaryngology and Dental Medicine	23 (0.4)	17 (0.3)	27 (0.5)	12 (0.8)	0.2405
Dermatology	17 (0.3)	22 (0.5)	25 (0.5)	2 (0.1)	0.3541
Allergy and Immunology	19 (0.3)	5 (0.1)	11 (0.2)	5 (0.3)	0.0036
Ophthalmology	3 (0.0)	4 (0.1)	23 (0.4)	4 (0.3)	0.6067

Percentages (in parentheses) indicate the percentage of total discharges at each site. *P* values represent results of chi-square tests comparing frequencies of conditions within each content category across sites

### Outcomes

We applied principal ICD-10 diagnosis codes from each site to the updated crosswalk tool to translate diagnosis codes into broad ABIM content categories and specific condition categories, yielding site-specific frequency distributions of clinical content seen by inpatient residents. Custom-written programs (developed using MATLAB; MathWorks Inc, Natick, Massachusetts) assigned ICD-10 codes to content categories and condition categories (codes available by request). The outcomes measured at each site include total number of discharges, number of captured diagnoses, and the number of discharges categorized by content and condition category.

### Analysis

Raw frequencies of content categories and condition categories were graphically represented by mosaic plots using the ‘R’ statistical programming environment (Version R 3.6.2). Such plots allow visual comparison of: a) relative frequency of each content category *within* each site (width of each box); and b) content frequency differences across each site (area of each box). Data were expressed as raw frequency, and not normalized to number of resident teams per site, to allow for aggregate program-wide characterization. N × 4 contingency tables were generated and chi-square tests employed to compare frequencies of condition categories across sites. For example, an 8 × 4 contingency table was generated to compare frequencies of each of the eight Allergy and Immunology condition categories across the four sites. P-values were corrected for multiple comparisons using the Bonferroni correction.

## Results

There were 18,604 total discharges from inpatient resident teams at NYU Internal Medicine Residency teaching hospitals in academic year 2020–2021 (6517 from NYU-BK, 5011 from NYU-MN, 5519 from BH and 1557 from VA). The crosswalk tool captured 6291/6517 (96.5%), 4866/5011 (97.1%), 5259/5519 (95.5%), and 1474/1557 (95%) of discharges from each site, respectively (Supplementary Table 2).

**Fig. 1:**
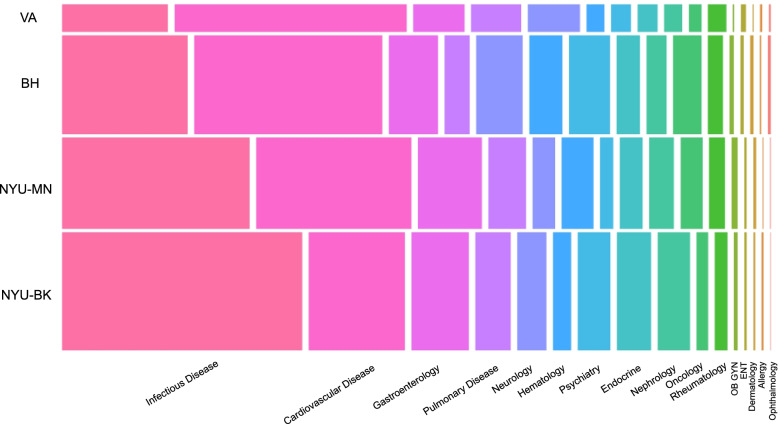
A mosaic plot depicting relative frequencies of patient discharges mapping to sixteen content categories across the four training hospitals of the NYU Internal Medicine Residency Program. The widths of each box reflect the relative frequency of content category within each site. Box areas reflect frequency of each content category relative to the other sites. NYU-BK (NYU Langone Hospital-Brooklyn), NYU-MN (NYU Langone Hospitals-Manhattan); BH (Bellevue Hospital). VA (VA NY Harbor Healthcare – Manhattan)

Infectious Disease (ID) and Cardiovascular Disease (CVD) were the two content categories seen with highest frequency at all sites. At NYU-BK and NYU-MN, ID predominated and represented 39.5% (*n* = 2485) and 30.8% (*n* = 1501) of total discharges at each site respectively. At BH and VA, CVD was the highest frequency content category and represented 31.0% (*n* = 1631) and 38.2% (*n* = 562) of discharges respectively (Fig. 1, Table 1). There were five content categories (Allergy and Immunology, Dermatology, Obstetrics and Gynecology, Ophthalmology, Otolaryngology and Dental Medicine) that represented less than 1% of total discharges at any site. (Fig. 1, Table 1).

There was substantial variability in the composition of content categories across the four sites. The distribution of conditions within ID was significantly different across sites (*p* < 0.001) (Table 1, Supplementary Table 2). Of note, Bacteremia and Sepsis Syndromes and Specific Causative Organisms, to which most COVID-19 related diagnoses were mapped, represented the highest frequency ID conditions at all sites (Supplementary Table 2). The distribution of conditions within CVD (*p* < 0.001), Endocrinology, Diabetes and Metabolism (*p* = 0.002), Gastroenterology (*p* < 0.001), Hematology (*p* < 0.001), Medical Oncology (*p* < 0.001), Nephrology/Urology (*p* < 0.001), Neurology (*p* < 0.001), Psychiatry (*p* < 0.001), Pulmonary Disease (*p* < 0.001), and Rheumatology/Orthopedics (*p* = 0.02) were significantly different across the four sites (Table 1, Supplementary Table 2).

## Discussion

Here, we leverage routinely-collected hospital data to provide a detailed characterization of IM residents’ inpatient clinical experiences at our program’s four diverse training hospitals over a full academic year. We unmask differences in clinical educational content across sites, categorized by both broad content categories and more specific condition categories. While it is unclear whether these findings are specific to our program or shared across IM training programs more broadly, we provide a reproducible strategy by which other programs can similarly map clinical data to better inform their trainees’ educational experiences, draw program-level comparisons, and drive evidence-based curricular change.

We demonstrate substantial differences in total inpatient volume to which residents are exposed across sites. Residents at NYU-BK, for example, participated in more total discharges during the academic year than residents at any other site, both in raw frequency and after accounting for the number of resident-staffed teams. These data have helped prompt important structural changes to the clinical learning environment at this site. For example, non-teaching services have undergone significant expansion, and resident team patient caps have been lowered to help create parity across sites and level clinical workload for residents.

Our program is notably enriched in relatively few content categories. ID and CVD content together comprise more than half of inpatient clinical experiences at each training site, and Gastroenterology and Pulmonary follow closely behind. Core content categories such as Rheumatology, Hematology, and Medical Oncology are consistently seen with less frequency at all four sites. These findings provide an opportunity to ‘rationally’ design curricula, whether explicit or experiential, to fill certain content gaps and perhaps complement high exposure content areas with specialized learning activities. Prior studies describe diverse approaches to using IM residents’ practice habits to inform curricular changes. Sequist and colleagues [10], for example, in their use of ICD-9 codes to classify outpatient IM clinical experiences, describe scheduling residents in subspecialty clinics to in an attempt to target underrepresented conditions. Mattana and colleagues [11], in addition to attempting to fill experiential content gaps, describe an approach to augment experiential learning with didactics in which residents were cohorted into content-based discussion groups based on the frequency with which they were exposed to content areas in clinical practice.

Our program has begun to harness these datain several different ways. NYU-BK residents were taken off of a month-long CVD-specific clinical rotation and instead are now scheduled for a two-week Hematology-focused clinical rotation and a two-week medicine-subspecialty rotation with flexible subspecialty offerings, including a Medical Oncology service. A Hematology and Medical Oncology didactic series focusing on those conditions least represented in clinical practice is being implemented in partnership with the fellowship program, and a similar series is planned for Rheumatology. New subspecialty outpatient rotations have been created at NYU-MN which provide the flexibility for residents to choose a subspecialty focus, among which several underrepresented content areas, notably Rheumatology, Nephrology, and Hematology will be offered.

While such curricular changes seek to balance exposure to content within each site, our findings also demonstrate that there are significant differences in the composition of content across sites. Not only are there differences in the overall frequency of CVD diagnoses across sites, for example, but there are significant differences in the distribution of conditions that comprise CVD across sites. In other words, the ‘type’ of CVD seen by residents differs across training sites, suggesting that there is a distinct CVD experience reflected by the hospital in which a resident trains; this goes for several other content categories as well, suggesting that there is real clinical individuality to our training sites that offer residents unique clinical experiences. A resident’s rotation schedule itself, which dictates how much clinical time is spent at each site, could form the basis for residents’ clinical strengths or weaknesses or even predict a predilection for a subspecialty focus for fellowship training. Importantly, this diversity can be harnessed; our program has begun to do so by scheduling rotations at sister sites (for example Manhattan-based residents rotating at the Brooklyn campus and vice versa) to provide more balanced clinical exposure. While this study purely profiles the clinical experiences of residents at each site, it forms the basis for future studies, underway at our institution, which will assess how differences in clinical exposure translate to differences in educational outcomes in our program, perhaps charting an experiential roadmap for success in residency.

Limitations of this study include the use of aggregate program-level, not individual-level, attribution of ICD-10 codes, which assumes residents within a given site share similar clinical experiences. The mapping strategy, which uses principal, and not secondary, ICD-10 diagnosis codes, intends to capture the singular compelling condition associated with hospital admission, however invariably ‘misses’ other conditions pertinent to hospitalized patients and thus to the education of residents. Future studies will address this by including select secondary ICD-10 codes, providing a more comprehensive mapping of clinical experiences. Given that exclusively inpatient, not outpatient, diagnoses are included here, it is possible that content deemed underrepresented in our study, such as Dermatology, Rheumatology, and Ophthalmology content, is in fact enriched in resident’s outpatient practice; further studies characterizing residents’ outpatient clinical experiences both in isolation and relative to inpatient experiences, will address this. Additionally, COVID-19 diagnoses were prevalent during the study period, likely skewing content frequency distributions toward ID content and limiting generalizability to a period of normalcy in which the pandemic is (hopefully) not as active.

## Conclusion

In this pilot, we translate discharge data from four distinct hospital systems into an educationally meaningful framework to characterize our residents’ educational experiences and in doing so unmask disparities in exposure that have driven rational curricular changes and can be expanded to other programs.

## Supplementary Information

**Additional file 1:** **Supplementary Table 1.** The completed crosswalk table anchoring 4959 ICD-10 diagnosis codes to 16 content categories and 177 condition categories.

**Additional file 2:** **Supplementary Table 2.** Frequencies of patient discharges mapping to each condition category across the four hospitals of the NYU Internal Medicine Residency Program. Total number of discharges, number of captured diagnoses, and the number of discharges categorized by content and condition category are shown.

**Additional file 3:** **Supplementary Table 3.** Principal ICD10 diagnosis codes of patients discharged from resident teams at each of the training hospitals of the NYU Internal Medicine Residency Program during the study period, which is divded into academic year quarter. Each tab corresponds to a single hospital's discharge data. NYU-BK (NYU Langone Hospital-Brooklyn), NYU-MN (NYU Langone Hospitals-Manhattan), BH (Bellevue Hospital), VA (VA-NY Harbor Healthcare-Manhattan). 

## Data Availability

All raw data analyzed for this study are included in this manuscript within the supplementary material (Supplementary Table 3).

## Abbreviations

GME: Graduate Medical Education
IM: Internal Medicine
ABIM: American Board of Internal Medicine
NYU-BK: NYU Langone Hospital – Brooklyn
NYU-MN: NYU Langone Hospitals – Manhattan
BH: Bellevue Hospital
VA: VA NY Harbor Healthcare – Manhattan
ID: Infectious Disease
CVD: Cardiovascular Disease
